# Husref Tahirović: Dr. Isak Samokovlija. The life in a white coat. Academy of Sciences and Arts of Bosnia and Herzegovina, Sarajevo, 2022

**DOI:** 10.17305/bb.2024.10816

**Published:** 2024-10-01

**Authors:** Ivan Damjanov

**Affiliations:** 1 Emeritus Professor of Pathology, The University of Kansas School of Medicine, Kansas City, KS, United States of America

Dr. Husref Tahirović, an endocrinologist and professor emeritus of pediatrics at the University of Tuzla School of Medicine and a member of the Academy of Sciences and Arts of Bosnia and Herzegovina, performed many functions and held many appointments during his long professional life, reflecting his various scientific and social interests. Among other roles, he has been the Editor-in-Chief of *Acta Medica Academica*, the official journal of the Academy. In this journal, he published several historical articles on famous physicians from Bosnia and Herzegovina. His interest in history also led him to spend considerable time during the last few years researching the life and works of Dr. Isak Samokovlija a celebrated writer in former Yugoslavia, who was also a physician recognized for his contributions to medicine and the organization of health care services in Bosnia and Herzegovina before the Second World War. This book contains a wealth of data that Dr. Tahirović has collected about this remarkable physician, who was born in Goražde in 1889 and died in Sarajevo in 1955.

In the history of literature of Bosnia and Herzegovina in the 20th century, Isak Samokovlija has a prominent place as an author of numerous short stories, some dramas, and even movie scenarios. Some literary critics have even lauded him as the founding father of Bosnian-Herzegovinian Jewish literature. Many reviewers of his creative writing mention that he was a practicing physician throughout his entire life. However, that important aspect of his life has somehow been neglected until now. In this book, Dr. Tahirović fills this gap, showing that the stories of this doctor-writer can be properly understood only in the context of his “other life”, i.e., the life of a medical doctor. One cannot ignore the fact that he was born to Jewish parents who raised their family in the small Bosnian town of Goražde, where Isak was born. But first and foremost, he was a member of the multiethnic Bosnian-Herzegovinian society, which he loved deeply.

It all began in the small Bosnian town Goražde, some 100 km from Sarajevo, where he was born into a Jewish family of small-town merchants. His mother moved with her school-aged children to Sarajevo so that they could receive a better education, while the father stayed in Goražde to mind the shop. With the financial support of the Jewish society *La Benevolencija,* he was sent to Vienna, where he graduated from the Medical School in 1917. After the war, he returned to his native town in Bosnia and began his medical practice. He was very busy providing medical assistance to the town folk as well as riding on horseback to help the rural population in neighboring villages. After a few years of practicing as a small-town and country doctor, he moved to Sarajevo, which he called his home until his death in 1955.

Dr. Tahirović chronicles Dr. Samokovlija’s daily medical practice between the two world wars, but even more dramatically, he describes the trials and tribulations of this highly respected Jewish doctor during the Second World War when Bosnia and Herzegovina became part of the Quisling Independent State of Croatia (NDH). In contrast to many other Jews who suffered considerably under the pro-German Croatian government, with many being deported or killed, Dr. Samokovlija and his family were relatively spared of most troubles due to his status as a physician, and he managed to survive the war unscathed.

He was frequently moved from one place to another, but in retrospect, this was most likely engineered by his medical colleagues in the government who tried to help him survive. During the war, he had to leave Sarajevo three times: the first time to attend compulsory army training in Ugljevik, after which he worked as a medical doctor taking care of the troops in that small town; the second time when he worked as an army physician: and the third time, in 1944, he was drafted to serve as a physician for the retreating Croatian and German armies. Upon reaching the city of Doboj, he managed to escape and join the partisans.

During the war, he worked as a physician on several major projects. He was instrumental in controlling the epidemic of typhoid fever in Sarajevo, which affected some 3000 inhabitants in 1942. He was also involved in fighting infectious diseases in the large refugee camp in Alipašin Most near Sarajevo. Officially, he was also part of the team fighting the spread of endemic syphilis in Banja Luka. As a member of that medical team, he was officially a government employee, which protected him and his family and helped him survive the horrors of the war.

After liberation, Dr. Samokovlija joined the Yugoslav National Army. When the war ended, he and his socially-minded friends, who were then in power, enthusiastically began organizing the health care services, which were almost nonexistent in that war-ravaged country. Recognized by his colleagues as a writer, he assumed the role of editor-in-chief of an educational medical magazine aimed at the populace at large. The magazine entitled *Life and Health*, had its first issue appeared in print in April 1946. It was printed in 20,000 copies and distributed free of charge to libraries, schools, and other public places. He edited the magazine for three years and wrote most of the educational articles.

The magazine was written primarily for lay readers, with most articles being stories about various diseases written in a colloquial, easy-to-understand format. Afterward, he became an editor at a major publishing company in Sarajevo, but still continued to practice medicine until the end of his life in 1955.

Dr. Tahirović has divided his book about Dr. Samokovlija into several parts. The first part deals with his life and medical practice, the medical education of common people, and the organization of health services. The second part contains a selection of medical educational articles and articles written for doctors and nurses reprinted from his magazine *Life and Health*. Reprinted are also nine poems about various diseases written by Dr. Samokovlija for the same magazine. At the end of the book, there is a complete list of his medical educational writings and all the photographs that Dr. Tahirović has collected to illustrate his book. A color portrait of Dr. Samokovlija painted by a well-known Bosnian-Herzegovinian artist, Mario Mikulić, currently in the JU Muzej Sarajeva (Museum of Sarajevo), is on the front page of the book.

In summary, this book is a valuable document about a remarkable man who was both a well-known writer and a physician. He lived in Bosnia and Herzegovina during some of the most turbulent times: two world wars separated by a 23-year-long interwar period, and an exciting postwar period of country rebuilding after 1945. Dr. Samokovlija left an indelible mark on both the cultural and medical history of his homeland, to which he devoted his entire life.

The book documents how he loved his countrymen and how they reciprocated that love in turn. Above all, this book is a document illustrating how love and dedication to Bosnia and Herzegovina and its people can surpass all troubles and also leave a deep mark on history.

